# Barking up the wrong biomarker? Correspondence to Shobeiri et al. (2022) “Serum and plasma levels of brain-derived neurotrophic factor in individuals with eating disorders (EDs): a systematic review and meta-analysis”

**DOI:** 10.1186/s40337-022-00662-2

**Published:** 2022-09-14

**Authors:** Jonas L. Steinhäuser, Marie-Louis Wronski, Johanna L. Keeler, Stefan Ehrlich, Joseph A. King

**Affiliations:** 1 Translational Developmental Neuroscience Section, Division of Psychological and Social Medicine and Developmental Neurosciences, Faculty of Medicine, Technische Universität Dresden, Dresden, Germany; 2 Section of Eating Disorders, Department of Psychological Medicine, Institute of Psychiatry, Psychology & Neuroscience, King’s College London, London, UK

**Keywords:** Anorexia nervosa, Bulimia nervosa, Binge-eating disorder, Eating disorders, Brain-derived neurotrophic factor, BDNF

## Abstract

Despite intensified research efforts into the underlying (neuro-)biology of eating disorders (EDs), only few reliable biomarkers of diagnostic or prognostic value have been identified to date. One promising line of research has focused on the role of peripheral blood-based biomarkers as potential contributors to the complex pathophysiology of EDs. One such candidate marker is brain-derived neurotrophic factor (BDNF), a neurotrophin broadly implicated in neuronal plasticity and food-intake regulation. A growing number of studies have targeted BDNF in EDs; culminating in several recent well-powered and controlled case–control studies, comprehensive meta-analyses, and review articles. In the current correspondence, we aim to put the recent meta-analysis of Shobeiri et al. (J Eat Disord 10(1):105, 2022) into perspective and argue that the finding suggestive of lower BDNF concentrations across individuals with EDs in comparison to healthy controls needs to be interpreted with caution. While this finding is compatible with those from earlier meta-analyses, it may be biased due to several reasons; most notably by the applied study selection procedures, insufficient consideration of influential determinants of BDNF concentrations, and generalization of results across the ED spectrum without sufficient statistical power. Further controlled and comprehensive studies are necessary to establish BDNF as a clinically informative biomarker of EDs.

We read with great interest Shobeiri and colleagues’ [1] recent review and meta-analysis of 14 studies on peripheral concentrations of brain-derived neurotrophic factor (BDNF) in females with anorexia nervosa (AN) and bulimia nervosa (BN) [1]. Across the studies included in the meta-analysis, Shobeiri et al. [1] found peripheral levels of BDNF to be lower in individuals with eating disorders (EDs) in comparison to healthy controls (HC). These meta-analytic results indicating the potential involvement of BDNF in the pathogenesis of EDs (indicative of a trait marker) and/or as an indicator of clinical response to therapy (indicative of a state marker) are important and will likely motivate and guide both research and clinical practice for years to come. Indeed, the authors boldly conclude from their findings that “*…interventions targeting the restoration of BDNF level to normal…*” would be beneficial in individuals with EDs.

## Inclusion of studies for the meta-analysis

However, although the study of Shobeiri et al. [1] has many strengths including state of the art meta-analytic methods and solid statistical analysis (e.g., leave-one-out analysis), we have some critical concerns regarding the study selection process, and consequently, the results obtained and the conclusions that can be drawn from them. Shobeiri et al. [1] state that studies were only included if they (a) were observational, measuring BDNF levels in individuals with EDs without other psychiatric or neurological comorbidities and (b) provided sufficient data, i.e., number of participants, mean, and standard deviation (SD) of BDNF levels. However, using the search criteria applied by Shobeiri et al. [1], we identified six studies that either met inclusion criteria [2, 3], would have met them if the authors had requested data from the investigators [4, 5], or simply calculated SDs from the reported statistical measures (i.e., 95% confidence intervals, interquartile ranges) [6, 7]. In fact, all of these studies were included in a recent meta-analysis of BDNF in AN that had similar inclusion criteria [8]. Moreover, some of the data from eligible studies could have also been obtained from a previous meta-analysis [9].

## Determining factors of BDNF in peripheral blood samples

Another issue that received insufficient consideration in Shobeiri et al. [1] relates to the challenges in the assessment and analysis of BDNF from peripheral blood samples. There are numerous determinants that influence BDNF levels and could drastically affect the results of BDNF group comparisons. First, the choice of blood component (i.e., serum or plasma) is highly relevant and might lead to measurement bias, given the higher sensitivity of plasma samples to pre-analytic decisions (e.g., choice of anticoagulant [10]) or handling of blood samples (e.g., pre-centrifugation delay [11]). Shobeiri and colleagues reported that the difference in BDNF levels between serum and plasma samples was not statistically significant. However, this absence of evidence contradicts methodological studies that found no correlation between serum and plasma BDNF samples [12] and even suggest that they reflect two different pools of BDNF [13]. Second, numerous studies have shown the influence of sample storage conditions (temperature and most importantly, storage time) on BDNF levels [7, 14–16]. It is possible that blood samples obtained from individuals with EDs might have a different storage time from HCs, depending on the duration of the study and availability of participants, resulting in altered BDNF concentrations in these specimens. This potential influence must be accounted for in investigations of BDNF and should have been discussed as a possible confound in the present meta-analysis. Third, physical activity has been shown to influence peripheral BDNF levels [17]. While it is difficult to have a standardized assessment of physical activity in clinical samples, exercise (e.g., as a purging mechanism) in patients with EDs may be a relevant confounding variable for peripheral BDNF concentrations. Lastly, following the notion that decreased levels of peripheral BDNF reflect a state of food-deprivation [18], it is critical to assess, evaluate, and discuss the fasting vs. non-fasting state of participants at time of blood draw. We acknowledge that it is not the responsibility of the authors of the meta-analysis to account for all of these methodological considerations. However, we strongly believe that these factors must be considered in the interpretation of these meta-analytic results and should be addressed in the discussion section of the manuscript.

## Generalizability of BDNF findings across eating disorders

We applaud Shobeiri and colleagues' aspiration to compare the role of BDNF across EDs, namely, AN, BN, and binge-eating disorder (BED). However, none of the studies they analyzed included any BED participants, limiting the interpretability of the results to AN and BN, which should have been communicated more transparently. More importantly, only two of the 14 included studies were conducted in BN specifically [19], one of which only included individuals that had been in remission for over six months [20]. Guidance from Cochrane [21] states that meta-analysis can be performed on two studies only, provided that their results are sufficiently similar. In the present investigation, the two studies included in the meta-analysis of BDNF concentrations in individuals with BN had opposite effect sizes (i.e., − 1.15 vs. 0.40) and included only small samples which reduces the overall statistical power. Especially when using random-effects models [22], it is essential that these limitations are stated in the Discussion to facilitate an accurate interpretation of the results. Further, Shobeiri and collaborators conducted a subgroup analysis comparing AN participants with BDNF concentrations measured in serum and BN participants with BDNF levels obtained from plasma samples, concluding that there was no significant difference in BDNF concentrations regarding the two blood components, or ED type. However, this conclusion cannot be drawn from the analysis conducted (Figure 2 in Shobeiri et al. [1]). Firstly, for the “AN and serum” subgroup they pooled studies that recruited both AN and BN participants (e.g., Nakazato et al. [23] that included 12 AN and 18 BN participants or Matsumoto et al. [24] that included 19 AN and 28 BN participants). Therefore, the “AN and serum” subgroup consisted of both individuals with AN and BN. This also precludes the ability to distinguish whether the absence of a significant difference is explained by the type of ED or the specimen used, as it appears that all studies included in the AN meta-analysis used serum specimens and all studies included in the BN meta-analysis used plasma specimens. Overall, the differentiation of how BDNF alterations might generalize across EDs as concluded by Shobeiri et al. [1] is very limited. Furthermore, the role of BDNF as an indicator of food-deprivation [18] aligns well with previous findings of decreased BDNF levels in AN of the restrictive, but not the binge/purge-type [8] and heterogenous results of BDNF levels in a systematic review in BN [25]. Together, these findings indicate that binging behavior might not result in, or be reflected by, decreased BDNF concentrations in BN and AN of the binge/purge-type. In light of these previous results and the limited informative value from the present meta-analysis of BDNF in BN, we are not convinced that there is substantial evidence for lower BDNF concentrations in BN, as concluded by Shobeiri et al. [1].

Lastly, Shobeiri and collaborators discuss their findings in light of previous reports on lower BDNF levels in depression [26], a frequent comorbidity in individuals with EDs [27]. They propose that lower BDNF levels serve as a risk factor for depression which in turn may increase risk of ED development. However, this hypothesis is not supported by their analysis, as Shobeiri et al. [1] explicitly excluded studies if ED participants had any psychiatric comorbidities. Further, this interpretation implies a role of BDNF would be a trait, rather than a state marker in EDs. This contradicts the fact that BDNF concentrations seem to rise over the course of treatment and return to normal (or even marginally higher) levels after recovery, as evidenced in a recent longitudinal meta-analysis of BDNF in AN [8], while depression scores remain at clinically significant levels even after weight-restoration [28]. This suggests that BDNF measures likely capture the acute state of illness rather than an aetiologic risk factor for the development of EDs.

## Conclusion

We thank Shobeiri and colleagues for their effort in advancing the field by providing new insight into the potential role of BDNF in EDs. Although these findings from studies mainly examining AN do not necessarily translate to BN and other binge-type EDs such as BED, they seem to underline the general notion of lower BDNF levels in AN as observed in individual studies and previous meta-analyses. Nevertheless, the comparatively small effect size implied by the available data suggests that BDNF might have little clinical utility in comparison to other candidate peripheral biomarkers including e.g., ghrelin, leptin, and gonadal hormones [29, 30]. We conclude that the meta-analysis of Shobeiri et al. [1] would have been more comprehensive by including additional relevant investigations of BDNF in ED populations [2–7] and we would be very keen to see if the findings are confirmed or changed by inclusion of these studies.

## Data Availability

Not applicable.

## Abbreviations

AN: Anorexia nervosa
BDNF: Brain-derived neurotrophic factor
BED: Binge-eating disorder
BN: Bulimia nervosa
ED: Eating disorders
HC: Healthy controls
SD: Standard deviation

## References

[1] Shobeiri P, Bagherieh S, Mirzayi P, Kalantari A, Mirmosayyeb O, Teixeira AL (2022). Serum and plasma levels of brain-derived neurotrophic factor in individuals with eating disorders (EDs): a systematic review and meta-analysis. J Eat Disord.

[2] Monteleone P, Fabrazzo M, Martiadis V, Serritella C, Pannuto M, Maj M (2005). Circulating brain-derived neurotrophic factor is decreased in women with anorexia and bulimia nervosa but not in women with binge-eating disorder: relationships to co-morbid depression, psychopathology and hormonal variables. Psychol Med.

[3] Saito S, Watanabe K, Hashimoto E, Saito T (2009). Low serum BDNF and food intake regulation: a possible new explanation of the pathophysiology of eating disorders. Prog Neuropsychopharmacol Biol Psychiatry.

[4] Ehrlich S, Salbach-Andrae H, Eckart S, Merle JV, Burghardt R, Pfeiffer E (2009). Serum brain-derived neurotrophic factor and peripheral indicators of the serotonin system in underweight and weight-recovered adolescent girls and women with anorexia nervosa. J Psychiatry Neurosci JPN.

[5] Mercader JM, Ribasés M, Gratacòs M, González JR, Bayés M, de Cid R (2007). Altered brain-derived neurotrophic factor blood levels and gene variability are associated with anorexia and bulimia. Genes Brain Behav.

[6] Dalton B, Campbell I, Chung R, Breen G, Schmidt U, Himmerich H (2018). Inflammatory markers in anorexia nervosa: an exploratory study. Nutrients.

[7] Steinhäuser JL, King JA, Tam FI, Seidel M, Biemann R, Wronski ML (2021). Is serum BDNF altered in acute, short- and long-term recovered restrictive type anorexia nervosa?. Nutrients.

[8] Keeler JL, Robinson L, Keeler-Schäffeler R, Dalton B, Treasure J, Himmerich H (2022). Growth factors in anorexia nervosa: a systematic review and meta-analysis of cross-sectional and longitudinal data. World J Biol Psychiatry.

[9] Brandys MK, Kas MJH, van Elburg AA, Campbell IC, Adan RAH (2011). A meta-analysis of circulating BDNF concentrations in anorexia nervosa. World J Biol Psychiatry.

[10] Engstad CS, Gutteberg TJ, Osterud B (1997). Modulation of blood cell activation by four commonly used anticoagulants. Thromb Haemost.

[11] Elfving B, Plougmann PH, Wegener G (2010). Detection of brain-derived neurotrophic factor (BDNF) in rat blood and brain preparations using ELISA: pitfalls and solutions. J Neurosci Methods.

[12] Tsuchimine S, Sugawara N, Ishioka M, Yasui-Furukori N (2014). Preanalysis storage conditions influence the measurement of brain-derived neurotrophic factor levels in peripheral blood. Neuropsychobiology.

[13] Gejl AK, Enevold C, Bugge A, Andersen MS, Nielsen CH, Andersen LB (2019). Associations between serum and plasma brain-derived neurotrophic factor and influence of storage time and centrifugation strategy. Sci Rep.

[14] Bus BAA, Molendijk ML, Penninx BJWH, Buitelaar JK, Kenis G, Prickaerts J (2011). Determinants of serum brain-derived neurotrophic factor. Psychoneuroendocrinology.

[15] Trajkovska V, Marcussen AB, Vinberg M, Hartvig P, Aznar S, Knudsen GM (2007). Measurements of brain-derived neurotrophic factor: methodological aspects and demographical data. Brain Res Bull.

[16] Zwipp J, Hass J, Schober I, Geisler D, Ritschel F, Seidel M (2014). Serum brain-derived neurotrophic factor and cognitive functioning in underweight, weight-recovered and partially weight-recovered females with anorexia nervosa. Prog Neuropsychopharmacol Biol Psychiatry.

[17] Huang T, Larsen KT, Ried-Larsen M, Møller NC, Andersen LB (2014). The effects of physical activity and exercise on brain-derived neurotrophic factor in healthy humans: a review: physical activity and BDNF. Scand J Med Sci Sports.

[18] Rios M (2013). BDNF and the central control of feeding: accidental bystander or essential player?. Trends Neurosci.

[19] Yamada H, Yoshimura C, Nakajima T, Nagata T (2012). Recovery of low plasma BDNF over the course of treatment among patients with bulimia nervosa. Psychiatry Res.

[20] Homan P, Grob S, Milos G, Schnyder U, Eckert A, Lang U (2015). The role of BDNF, leptin, and catecholamines in reward learning in bulimia nervosa. Int J Neuropsychopharmacol.

[21] McKenzie J, Brennan S, Ryan R, Thomson H, Johnston R. Chapter 9: Summarizing study characteristics and preparing for synthesis. In: Higgins JPT, Thomas J, Chandler J, Cumpston M, Li T, Page MJ, Welch VA, editors. Cochrane Handbook for Systematic Reviews of Interventions version 63 (updated February 2022) [Internet]. Cochrane; 2022. Available from: www.training.cochrane.org/handbook.

[22] Borenstein M, Hedges LV, Higgins JPT, Rothstein HR (2009). Introduction to meta-analysis.

[23] Nakazato M, Hashimoto K, Shimizu E, Kumakiri C, Koizumi H, Okamura N (2003). Decreased levels of serum brain-derived neurotrophic factor in female patients with eating disorders. Biol Psychiatry.

[24] Matsumoto J, Hirano Y, Hashimoto K, Ishima T, Kanahara N, Niitsu T (2017). Altered serum level of matrix metalloproteinase-9 and its association with decision-making in eating disorders: MMP-9 and decision-making in ED. Psychiatry Clin Neurosci.

[25] Phillips K, Keane K, Wolfe BE (2014). Peripheral brain derived neurotrophic factor (BDNF) in bulimia nervosa: a systematic review. Arch Psychiatr Nurs.

[26] Sen S, Duman R, Sanacora G (2008). Serum brain-derived neurotrophic factor, depression, and antidepressant medications: meta-analyses and implications. Biol Psychiatry.

[27] Herzog DB, Nussbaum KM, Marmor AK (1996). Comorbidity and outcome in eating disorders. Psychiatr Clin North Am.

[28] Tomba E, Tecuta L, Crocetti E, Squarcio F, Tomei G (2019). Residual eating disorder symptoms and clinical features in remitted and recovered eating disorder patients: a systematic review with meta-analysis. Int J Eat Disord.

[29] Berner LA, Brown TA, Lavender JM, Lopez E, Wierenga CE, Kaye WH (2019). Neuroendocrinology of reward in anorexia nervosa and bulimia nervosa: beyond leptin and ghrelin. Mol Cell Endocrinol.

[30] Malcolm A, Phillipou A (2021). Current directions in biomarkers and endophenotypes for anorexia nervosa: a scoping review. J Psychiatr Res.

